# Implementation of tele visit healthcare services triggered by the COVID-19 emergency: the Trentino Province experience

**DOI:** 10.1007/s10389-021-01609-8

**Published:** 2021-06-15

**Authors:** Sara Testa, Oscar Mayora-Ibarra, Enrico Maria Piras, Olivia Balagna, Stefano Micocci, Alberto Zanutto, Stefano Forti, Diego Conforti, Andrea Nicolini, Giulia Malfatti, Monica Moz, Lorenzo Gios, Pier Paolo Benetollo, Ettore Turra, Massimo Orrasch, Francesca Zambotti, Maurizio Del Greco, Massimiliano Maines, Lorena Filippi, Monica Ghezzi, Federica Romanelli, Elisabetta Racano, Mauro Marin, Marta Betta, Elisabetta Bertagnolli

**Affiliations:** 1 Fondazione Bruno Kessler, Via Santa Croce 77, 38122 Trento, Italy; 2 Provincia Autonoma di Trento, Piazza Dante 15, 38122 Trento, Italy; 3 Azienda Provinciale per i Servizi Sanitari, Via Alcide Degasperi 79, 38123 Trento, Italy

**Keywords:** Telemedicine, Tele visit, Tele consult, eHealth, COVID-19, Digital health

## Abstract

**Aim:**

In response to the SARS-CoV-2 emergency, the Competence Centre on digital health ‘TrentinoSalute4.0’ has developed TreC_Televisita, a tele visit solution that meets the needs of the Trentino healthcare system and maintains high-quality patient–doctor interactions while respecting social distancing. This paper highlights how ‘TreC_Televisita’ was integrated into the Trentino healthcare system and its potential to become a structural and durable solution for the future local healthcare service provisioning.

**Subject and methods:**

This paper presents the multifactorial context that TreC_Televisita has faced for its implementation and the strategies adopted for its structural integration into the healthcare system. The analysis focuses on the main issues faced for the integration of the tele visits (e.g. privacy, payments) and how the context of TrentinoSalute4.0 permitted responding quickly to its implementation during the pandemic. It also describes how TreC_Televisita fits into the healthcare continuum from the organisational and technological standpoint, the end-user perspective and the barriers that could hamper the solution scalability.

**Results:**

TreC_Televisita has demonstrated to be a technological solution that can be contextualised for different clinical domains beyond SARS-CoV-2. Moreover, it has shown its potential to scale up the solution beyond the COVID-19 emergency to the whole healthcare provisioning system in the long term.

**Conclusion:**

Being a positive experience in the first months of its implementation, the long-term goal is to transform TreC_Televisita into a structural pillar of the Trentino healthcare system, setting the bases for a sustainable, win–win situation for all the stakeholders involved in healthcare service provisioning.

## Introduction

The novel coronavirus disease, COVID-19, was first detected in China in December 2019 and, since then, it has spread worldwide; almost all healthcare systems throughout the world have been severely affected by such a heavy burden. In Europe, Italy was the first country hit by this disease and one of the most suffering. In late February 2020, the Italian Government imposed a lockdown to the citizens in order to ensure social distancing, which is considered one of the most effective – albeit demanding – preventive measures to halt the virus propagation. A great part of the Italian healthcare staff was particularly under pressure for the management of COVID-19 patients, and especially for the dramatic high number of those hospitalised in intensive care units; nonetheless, local healthcare trusts also had to keep providing assistance to citizens despite social distancing. In this scenario, the Healthcare Trust of the Autonomous Province of Trento – Azienda Provinciale per i Servizi Sanitari (APSS) – identified as a main priority the setup of a solution for the remote provisioning of healthcare services to allow reducing the risk of contracting the virus and to comply with mobility restrictions. For this purpose, a remote service to connect the healthcare staff and the patients was implemented adopting a systematic tele visit approach. Tele visit has been defined by the Italian Ministry of Health as the ‘health-related act of remote interaction between doctor and patient^1^’, and by the World Health Organization (WHO) as ‘the delivery of health care services, where distance is a critical factor, by all health care professionals using information and communication technologies for the exchange of valid information for diagnosis, treatment and prevention of disease and injuries’ (WHO 2009).

This paper focuses on the implementation of a tele visit service in the Autonomous Province of Trento, Italy. Before the COVID-19 emergency, such a system was not available in the Province: it was only planned as a future development to improve the healthcare provisioning service for better serving the citizens located far from the main healthcare facilities in Trento. The pandemic stressed the need for implementing such a service quickly to overcome the limitations imposed by the emergency in the whole territory. Therefore, tele visit was chosen precisely because it has been already explored in other regions worldwide where remote assistance is necessary and has been well documented in literature and acknowledged by public administrations and healthcare institutions. The main challenge faced by the Province of Trento in relation to the tele visit was its rapid and effective implementation, and this has been the main driver for the research question object of this work. This paper analyses how tele visit could be integrated into the healthcare system from an organisational and technological point of view in a short period of time and which aspects should be considered in order to scale it up into a structural service of the Trentino healthcare system in the longer term. In addition, the paper highlights the various barriers encountered for the implementation of the proposed solution (organisational, technical, ethical and legal) that can be taken into consideration as a precedent for the immediate and long-term implementation of similar systems in other geographical contexts.

The paper is organised as follows. First, it frames the context of digital healthcare services provisioning in the Province of Trento through TrentinoSalute4.0 (TS4.0) Competence Centre. Then, it describes the methodological framework adopted for TreC_Televisita: this includes the analysis of the elements necessary for developing and integrating the service by eliciting the organisational and technical requirements as well as the regulatory ones, an analysis of the outcomes obtained after the preliminary user evaluations conducted in a living lab environment, and of the possible barriers for the structural inclusion of the service in the healthcare system beyond the COVID-19 contingency period. Finally, the results of the TreC_Televisita projects are analysed, and the related conclusions are drawn.

### Background and overall context of TreC_Televisita

**Fig. 1:**
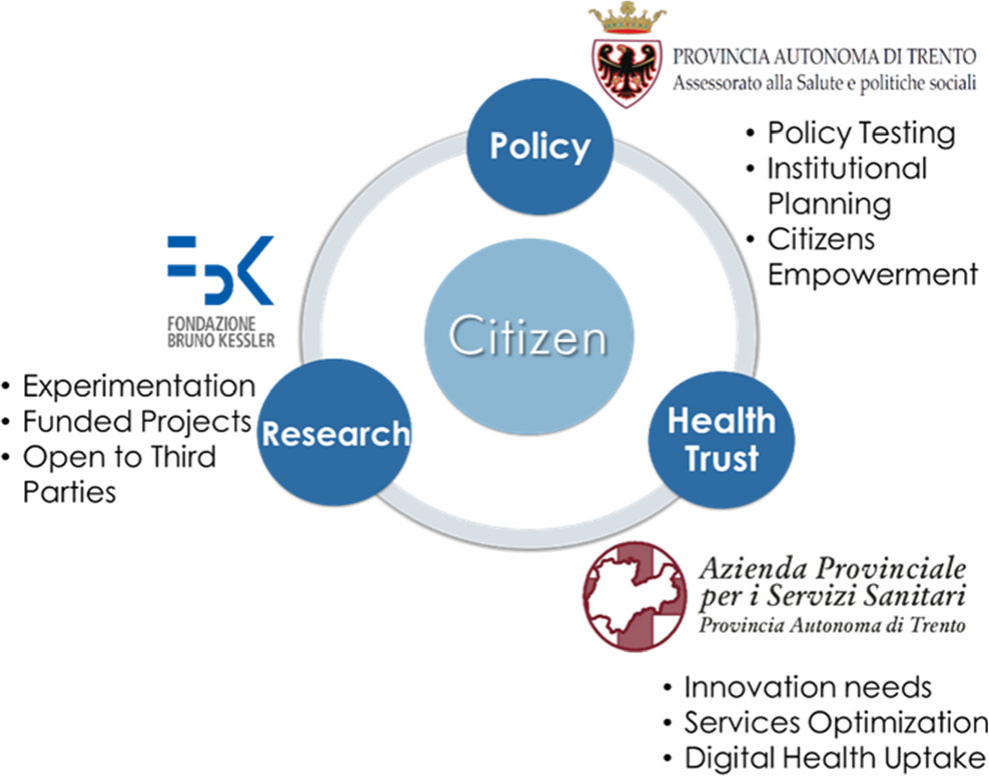
TrentinoSalute4.0 stakeholders and main roles

TreC_Televisita has been realised through the support of the TS4.0 Competence Centre on Digital Health of the Autonomous Province of Trento. TS4.0 was formally established with an Act of the Local Government n. 2412 on 20 December 2016: the partnership governance includes the Autonomous Province of Trento (PAT) through the Department of Health and Social Policies in the role of decision-maker, the local Healthcare Trust (APSS) in the role of the health service provider and the Bruno Kessler Foundation (FBK) as the research institute responsible for technological innovation, as shown in Fig. 1. TS4.0 also involves citizens, health professionals and sector companies according to a quadruple helix model (Mayora-Ibarra et al. 2019). Also, in May 2020, TS4.0 has officially become a Joint Research Unit for strengthening cooperation among the institutions. This kind of virtuous partnership has been considered a good practice enhancing sustainability in healthcare provisioning (Botti and Monda 2020).

As the key local actor in digital health for Trentino Province, TS4.0 had the mandate to support the development and adoption of digital solutions to quickly respond to the needs that emerged during the COVID-19 lockdown. TS4.0 fast reaction was possible thanks to the long-term strategic cooperation on digital health among the three main stakeholders that have worked side by side since 2017, leading to a swift and robust decision-making process. In fact, this relationship has led to the increase of the digitalised services offered to the citizens in the Trentino Province, including both those strictly integrated with the healthcare system, which feature a medical dashboard and an app for the patients such as TreC_Diabetes (Dragoni et al. 2019) TreC_Cardio (Maines et al. 2020), and those offered to the citizens for health promotion (e.g. Salute +, an app based on gamification promoting healthy lifestyles).

All the digital services provided by TS4.0 are sustained by the ‘TreC’ (the Trentino Citizens Clinical Record) platform, which is the personal health record (PHR) of all Trentino citizens (Eccher et al. 2020). TreC has been developed as a modular platform whose extensible architecture allows sub-systems to be integrated for the provision of additional and specific functionalities (Osmani et al. 2017). Therefore, TreC has become the pillar for the long-term strategy of healthcare digitalisation – a ‘one-stop-shop’ for the Trentino healthcare system – as, beyond its function as PHR, it also provides a place for testing new technologies. Moreover, TreC can include third party apps and microservices, and is compliant from the legal and ethical viewpoints (e.g. GDPR). During the COVID-19 emergency, TS4.0 was able to ensure the development of new apps to respond quickly to the emergency, that is, TreCovid^2^ (Gios et al. 2021) and TreC_Televisita. The existence of one healthcare provider for the whole Trentino territory has the advantage of having a unique PHR where all patients’ data are gathered. In addition, because the whole digital healthcare system in Trentino is built into TreC, it is currently used by a large number of the Trentino population (over 110,000 users) which facilitate the scaling-up of new digital services and applications, such as TreC_Televisita.

The production of digital solutions for positively facing the COVID-19 situation in Trentino over the TreC platform was done in two steps. The first solution implemented during the COVID-19 emergency was the TreCOVID-19 app, released on 16 March 2020. The app had a double role: on the one hand, it supported the citizens in better understanding the initial rules during the lockdown with reliable and official information for facing the pandemic. On the other hand, it provided a home monitoring infrastructure for diagnosed and suspicious COVID-19 patients to collect their physiological and behavioural parameters related to the disease and transmit them to the doctors for supporting treatment decision-making. The second step was the implementation of the TreC_Televisita solution, conceived from the alignment of patients and healthcare professionals needs on the one side, and healthcare and policy management requisites on the other. From the patients and practitioners’ side, the request for the implementation of tele visits did not emerge during the COVID-19 emergency but started a few years ago: in the Trentino Province, the two main healthcare districts (located in Trento and Rovereto) are not easily accessible to citizens living in the faraway valleys, where a trip to the main cities can take up to one and a half hour driving or more than a two-hour journey with public transport. For example, the need for tele visits clearly emerged from the interviews conducted during an early pilot activity conducted back in 2018 for remote monitoring of pregestational diabetes patients (Piras and Miele 2019).^3^ Since March 2020, the COVID-19 emergency has triggered the healthcare management and policy-makers to decide to implement tele visits as a way to mitigate, on the one hand, the issue of accessing healthcare services due to mobility limitations and, on the other, as a means to reach distant communities, such as the numerous valley communities of Trentino. The need for tele visits became officially a political priority through a formal action of the Autonomous Province of Trento, namely the deliberation of the Provincial Council n°456 from 9 April 2020, called ‘Provisions on Telemedicine and other provisions to deal with the emergency from COVID-19’. With this resolution,^4^ the Provincial Council has updated the nomenclator of outpatient specialist assistance services, of diagnostic imaging and laboratory specialists by integrating it with telemedicine services (services provided remotely). In this way, the Autonomous Province of Trento was the first of the Italian Regions/Provinces to provide telemedicine in the era of COVID-19 as an alternative to the traditional healthcare approach. From the healthcare–provisioning side, in March 2020, i.e. after the mobility restrictions were imposed, the need to substitute a service traditionally performed on-site, i.e. patient–clinicians face-to-face visits, led to the decision of TS4.0 management of developing TreC_Televisita solution.

### Methodology

The TreC_Televisita project has its basis on a multidisciplinary approach that is intrinsic to the TS4.0 partnership by integrating clinical, technological and policy-related expertise, with a focus on implementation research. The methodology implemented in TS4.0 stands on evolving research developments with medium technology readiness levels (TRL 5–7) into validated systems and services with TRL8 and 9 with the goal of ameliorating public health outcomes, scalability and sustainability of solutions for improving citizens’ health, as suggested by Theobald (Theobald et al. 2018). An inherent characteristic of TS4.0 implementation research methodology is that the approach chosen is not based on various context elements such as the vertical (disease-specific) or horizontal (cross-cutting) focus: rather, it shifts the attention towards the links between research and practice, and the different actors and competences involved. The implementation research approach utilised by TS4.0 considers a real-world setting, often without the specificities and restrictions of a clinical trial and allows a real-time adjustment of the solutions in an iterative and dynamic process (Theobald et al. 2018; Peters et al. 2013). This methodological approach was adopted for TreC_Televisita as it has demonstrated to be effective for the definition of the other services already integrated into TreC, e.g. TreC_Diabetes (Eccher et al. 2020).

Therefore, despite the unexpectedness of the situation in the spring of 2020, the realisation of TreC_Televisita was the natural – albeit accelerated – prosecution of the work of TS4.0 in the provisioning of eHealth services for the Trentino citizens and its development followed the same approach. Such methodological approach has been implemented with three different steps:

i. Identification of App requirements – The first step of the methodology for the app realisation is the gathering of the organisational and technological requirements, meaning those elements necessary for the integration within the healthcare process and clinical procedures and for embedding the service into the existing TreC platform (Piras et al. 2010). For the organisational requirements, the whole patient management process was considered, starting when the patient is taken in charge by the doctor, until the monitoring after the (tele) visit. Considering the time-constraints for delivering the solution, we have first analysed the requirements gathered for telemonitoring past solutions (e.g. TreC_Diabetes), adapted them to tele visits and, as a final step, these were validated with healthcare professionals and modified when needed.
ii. Compliance with regulatory issues – The second phase focused on the structural issues related to its implementation because the TreC_Televisita overall solution needed first to comply with the framework of the Trentino Healthcare provisioning system in terms of payment methods, data protection and validity assessment. Data protection issues followed a privacy-by-design approach developed together with the healthcare staff to define, for instance, the information sheets. Validity assessment and payment methods were dealt with at a management level within the policy entity and the healthcare trust.
iii. User evaluation in a living lab environment – For TreC_Televisita, four case studies were identified with the aim to test feasibility related to various users’ aspects such as usability and perceived usefulness. A mixed-method approach was adopted to investigate the organisational impact, the patient–provider relationship, and the solution usability: the final objective was to gather a generalisable understanding drawing on theoretically selected cases. For the evaluation of usability, initially a heuristic evaluation by user-interface researchers was conducted and successively a cognitive walkthrough exercise assigning specific tasks during a simulated tele visit to healthy volunteers.

Owing to the contextual factors already described in the first part of the paper (i.e. the centralisation of the healthcare infrastructure in Trento, its Alpine morphology and the related complex transportation network), which makes Trentino a particularly suitable environment for the exploitation and scalability of the TreC_Televisita service, the methodology has been enriched with a fourth step. This fourth step follows the implementation research approach defined by Peters (Peters et al. 2013), which underlines that the investment of money that focuses on the health innovation per se rather than its exploitation, too often leads to a failure when it comes to the shift from small-scale piloting to large-scale implementation. Therefore, an additional component was considered, namely:

iv. Future sustainability – the analysis for the longer-term applicability of the TreC_Televisita service has been considered since its conception for addressing both: the immediate needs originated by the pandemic, and the identification of the barriers for the long-term sustainability of the solution together with the strategy for overcoming them. The state-of-the-art frameworks concerning the identification of barriers to telemedicine implementation were analysed: the framework that better matched the Trentino context was used as the starting point for the definition of the potential solutions and lessons learnt.

The methodology applied, as well as the analysis of the results and the lessons learnt, could serve as a starting point for other healthcare systems towards the definition of tele visits: in particular, the app requirements for both the organisational and the technological infrastructure as well as the necessary elements for the compliance with ethical and legal issues could be scaled up to other contexts, especially in the European Union. In addition, the long-term sustainability section provides a framework that is adaptable to different context and can be the basis for a feasibility study for the long-lasting implementation of new services into healthcare systems.

### Identification of app requirements for the integration in the Trentino healthcare process

As explained above, the first step of the methodology is the identification of the requirements for the development of the app: TreC_Televisita is a digital solution integrated into the healthcare provisioning system of Trentino Province; thus, it should be part of a patient’s path when taken in charge by the doctor. For this purpose, a series of procedural changes needed for the correct implementation of the tele visit were identified. These procedural changes included not only the tele visit itself but also pre and post actions to be performed by the patient after the prescription of this intervention. In the first phase, the preliminary visits held remotely during the COVID-19 emergency were conducted for eliciting overall organisational requirements of the solution by implementing existing technologies based on phone calls and the Google ‘Meet’ App. A successive version implemented a mobile app for patients and a front-end web dashboard for the healthcare staff supporting clinical professionals for prescribing the Trec_Televisita app, scheduling tele visits, and checking patient’s data; the functionalities of the patients’ app included inserting files, images, chatting, viewing scheduled appointments, video calling, etc.

**Fig. 2:**
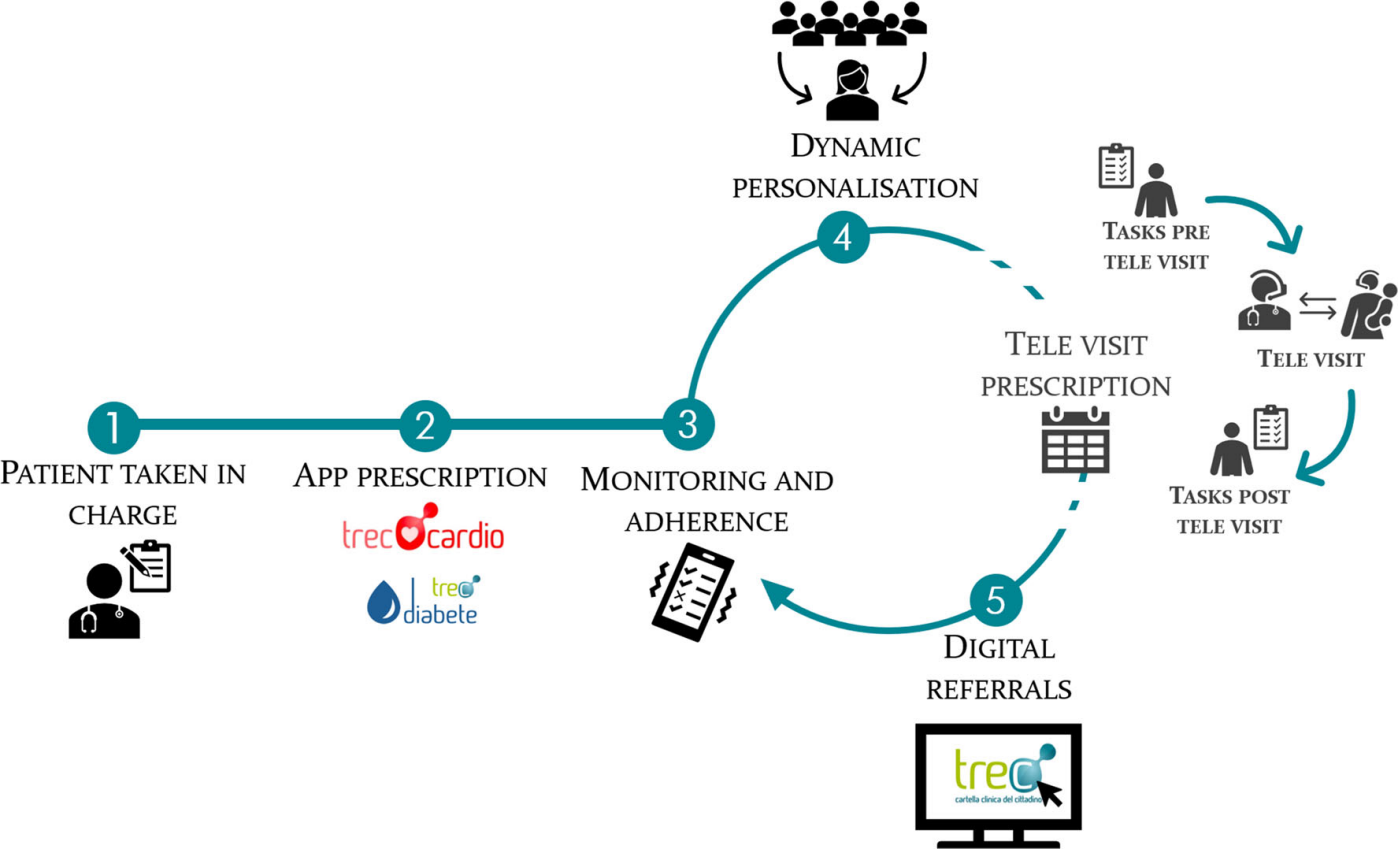
TreC_Televisita as part of digital management of health condition

Since the lockdown period, different healthcare domains have adopted various instances of the TreC_Televisita service in Trentino besides those managing COVID-19 patients. Amongst the first ones, diabetologists and cardiologists requested its use for overcoming some of the collateral problems raised by the pandemic (Maines et al. 2020). Additionally, ophthalmologists, paediatricians and occupational doctors voluntarily requested the provisioning of tele visits for their patients in an experimental way. From mid-April until the end of July 2020, a total of 12,000 tele visits were performed for the above-mentioned diseases’ domains. It is important to mention that while the tele visit service is conceived as a standard conceptualisation for all diseases, each domain visit requires ad-hoc customisation to adapt the remote consultation services and specific requirements. In this regard, the features implemented for each clinical speciality are different, as TreC_Televisita is a solution adaptable to the needs of each domain. For example, ophthalmologists explicitly requested the possibility for their patients to upload specific information to the portal (e.g. images, pdfs) before the consultation, as a prerequisite for conducting a tele visit. In this way, TreC_Televisita manages to follow the different necessary customisations according to the disease-specific procedures and requirements following the general outline described in Figs. 2. Figure 2 shows how the tele visit prescription may be optionally activated between step 4 and 5 of the telemonitoring process.

Figure 2 displays the path a patient will follow when they enter the healthcare system and is supported by digital health technologies for a specific condition: as an example, the path followed by a diabetes patient is described. First, the general practitioner (GP) prescribes the patient a visit in the diabetology centre, where the patient is taken in charge by the diabetologist (step 1): after a careful examination of the symptoms, habits, etc., the diabetologist prescribes an app – i.e. TreC_Diabetes, and a personalised care plan (step 2). During the weeks after the visit, the patient’s adherence to the treatment is monitored (step 3) supported by the TreC Diabetes virtual coaching platform (Maimone et al. 2018). On the medical dashboard, the diabetologist can view the patient’s data on a regular basis and modify the treatment following a step-based approach in order to increase the patient’s adherence (step 4). When needed, the doctor can prescribe the patient a tele visit and other exams to perform before the visit and upload the results (pdf, images, etc.) on the tele visit system: these tests will be examined by the doctor during the tele visit in order to have an even more complete clinical picture of the patient. Afterwards, the doctor might assign additional tasks (e.g. diet, drug prescription) to the patient. The results of the tele visit and other monitoring tasks will generate specific referrals. The generation of these referrals will be further achieved in successive versions of TreC_Televisita by automatically updating them from TreC PHR. From this step, the healthcare continuum cycle reconnects with step 3, where the doctor monitors the patient’s compliance.

While the previous flow of events is the same for most of the digital treatments of the TreC ecosystem, the tele visit component is only included if a patient or their clinician requests it. Moreover, in the case of COVID-19 management during the lockdown, the tele visit component operated independently from the 5-step-flow as it is prescribed directly to patients at risk.

Regarding reimbursement and payment management of tele visit service during the pandemic, the Province of Trento decided to apply a solution that could simplify the wider access to this kind of service to the population. In non-pandemic scenarios, the prescribed visit is associated with a code corresponding either to a payment or the partial or full exemption. The Trentino healthcare system had never faced the issue of payment associated to tele visits; however, payment and reimbursement have been a sensitive issue for many years in the tele visit context. In fact, this has been constantly evolving since the early 1990s when in some countries, such as the United States, telemedicine provision was excluded from the Medicare programme as it explicitly required ‘processes ordinarily involving physician–patient contact be *delivered in person*’. This changed in 1999 when Medicare increased both the number of telehealth services covered and payment rates for these services (Gilman et al. Gilman and Stensland 2013). Payment parity was not ensured for the same service code, representing a hurdle for exploiting the full potential of tele visits, until COVID-19 (Shachar et al. 2020); however, in order to ensure widespread implementation of tele visits, it would be necessary to maintain this equity even after the pandemic. In this regard, during the pandemic and until the end of the state of emergency, the Autonomous Province of Trento declared that the services of telemedicine (including tele visits) would be provided in the scheme of exemption from sharing health care expenses (so-called ticket) for all patients registered with the national health service, specifying that this exemption, (identified with the ‘TEL’ code), applies regardless of the assessment of the COVID-19 contagion.

### Identification of app requirements: TreC_Televisita technical integration

**Fig. 3:**
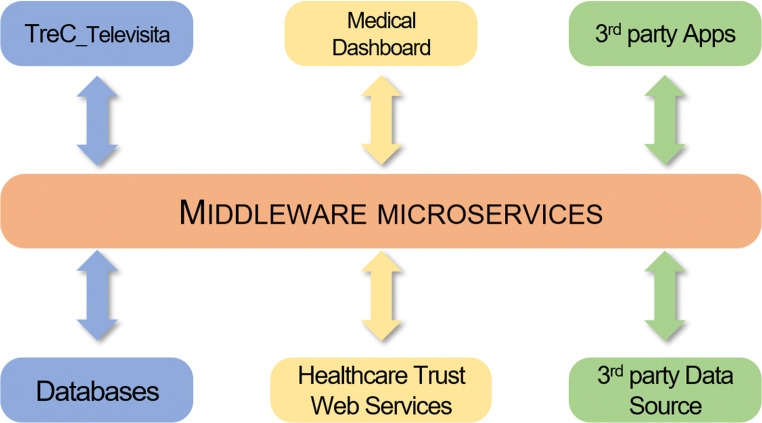
TreC_Televisita implementation within the TreC ecosystem

TreC_Televisita provides an environment for testing and implementing innovative data-driven telemedicine services. Originally, it was conceived to mitigate COVID-19 emergency and later-on to extend its functions for post-lockdown services to other disease domains. The TreC_Televisita system incorporates various services through the TreC middleware that interconnects a series of sensors and devices from multiple data sources (such as EHRs, apps) in order to allow the combination of their data flows either for immediate decision-making of clinical professionals on treatment management or for other processing purposes, i.e. for virtual coaching services relying on previously developed systems (Fig. 3) (Maimone et al. 2018; Dragoni et al. 2018). Moreover, other basic services such as authentication and access control are already provided by the TreC ecosystem. Other specific functionalities such as the telemonitoring hub and dedicated messaging system are also provided through the TreC middleware interconnecting the TreC_Televisita app with the medical dashboard. In this way, the tele visit system allows the prescription and intervention of a clinician through the medical dashboard functionality for any further required direct patient–clinician contact.

In order to define all steps related to handling the remote visits, TreC_Televisita utilises the disease management workflow and knowledge from the respective approved protocols and good practices for handling each disease. The TreC_Televisita technological infrastructure is constantly monitored to ensure its proper functioning and its protection from possible cyber-attacks.

### The compliance of TreC_Televisita with regulatory issues

Notwithstanding the boost of tele visits performed globally during the COVID-19 emergency, some formal issues connected with their implementation remain unsolved, especially in a long-term perspective; these will need to be determined by Governments more structurally in the longer term in order to ensure the structural integration of tele visit in the healthcare provisioning system (Romanick-Schmiedl et al. Romanick-Schmiedl and Raghu 2020). In the meantime, because of the urgent need for coping with the emergency situation in many regions, basic aspects related to the use of technology within the healthcare sector (e.g. privacy) were deregulated. The following paragraphs analyse the second step of the methodology corresponding to the identification of the issues that the Trentino healthcare system had to comply with for the implementation of the TreC_Televisita solution: these are personal data protection and validity assessment.

The management of personal data has always been a crucial issue when implementing eHealth solutions (Robles et al. 2020), also within the Trentino healthcare system. In the EU, all the services related to telemedicine, scientific research,^5^ the use of apps for social distancing measures, warning and contact tracing^6^ needed to comply with the current legislation, e.g. the General Data Protection Regulation (Regulation (EU) 2016/679) – GDPR. In particular, according to article 9, the processing of health-related and genetic data is prohibited, but it is allowed^7^ when (art. 9.2.g) ‘processing is necessary for reasons of substantial public interest’ and when (9.2.j) ‘processing is necessary for archiving purposes in the public interest, scientific or historical research purposes or statistical purposes’. Moreover, the article states that ‘processing shall be proportionate to the aim pursued, respect the essence of the right to data protection and provide for suitable and specific measures to safeguard the fundamental rights and the interests of the data subject^8^’. For the implementation of TreC_Televisita, the aspects related to personal data management in the Trentino Province were considered by design thanks to the inputs from a multi-professional group of experts. Ad hoc documents (including information sheets for citizens/patients) have been developed in line with the privacy regulation’s principles, whilst from a structural viewpoint, tele visits’ tools and systems were embedded in the PHR platform, which has been developed in line with the General Data Protection Regulation standards.

Considering the lack of time available for a clinical trial phase for the validation of the TreC_Televisita service, another important issue to consider related to the implementation of tele visits is the validity assessment, meaning the quality and the effectiveness of the service compared to the traditional face-to-face practice. Literature has underscored that traditional visit and tele visit can be comparable: for example, for neurological care (Bloem et al. 2020) videoconferencing was even deemed more efficient, while home activity-based training for survivors of stroke was equally effective. However, the randomised trials aimed at assessing tele visit quality mostly focused on chronic patients with stable conditions and some studies have shown the concerns of clinicians related to clinical quality as well as accountability and, thus, insurance issues (Greenhalgh et al. 2020; Wherton et al. 2020). In Italy, the clinicians association (FNOMCEO) has criticised^9^ the proposal^10^ of the Health Commission of the Italian Regions and Autonomous Provinces related to the implementation of tele visit during the pandemic: article 87 of the Clinicians’ Deontological Code explicitly mentions that tele visit *cannot* substitute the direct doctor–patient relationship but can be used for remote monitoring, and article 24 states that a certification of the patient’s health status is the consequence of a *direct* patient’s evaluation. While the previous considerations apply widely in Italy, the experience in Trentino Province in this regard can be considered unique. Significant and concerted efforts have been promoted by the local Health Trust (APSS) to develop and to standardise a set of procedures to ensure proper implementation and management of tele visits during the epidemic acute phase, covering a large number of clinical activities from first consultations to follow up visits. After the infection’s peak, the core procedures have been strategically adapted to cover follow up sessions and tele consultations. The entire process has been promoted, coordinated and monitored by multidisciplinary groups of experts, to guarantee smooth implementation and response to the fast-changing epidemiological situation.

### TreC_Televisita evaluation in a living lab environment

In the previous sections, we have illustrated the institutional framing of TreC_Televisita as a part of the services offered by the local Healthcare Trust and some technical specifications of the platform. While these aspects are highly relevant, it is important to verify that they do not only reflect the perceived needs of the institutions but also those of the healthcare providers and patients, the end-users of the platform. In line with previous experiences with remote monitoring based on the TreC Platform (Piras and Miele 2017; Piras and Zanutto 2014; Passardi et al. 2017) we have piloted and evaluated TreC televisita in a living lab environment to address organizational and acceptability issues.

For the sake of this article, which intends to provide the full picture of the institutional framing, the technical design and piloting implementation of TreC_Televisita, we shall limit the description of the methodology and results of the case study analysis which requires a paper in its own right. From its conception, the TreC_Televisita project had the ambitious goal to define a standard for a larger set of clinical practices to cope with the problem of social-distancing while providing a solution for clinicians to follow their patients: this solution can also be exploited for tele visits with COVID-19 patients who are, at the moment, monitored with the TreCOVID-19 app. To this end, the chosen approach was to proceed with four case studies whose selection was theoretically informed according to two axes: routine visits vs. acute care visits, structured vs. open-ended visits. The two axes allow identifying four broad categories of reasons for encounter that can be applied, by analogy, to many different consultations: routine visits structured around a specific health issue, routine control visits, visits triggered by an acute need that requires monitoring over time, and visits needed to evaluate an unspecified acute need. Table 1 below presents the selected cases studies.

**Table 1:** Case studies – healthcare professionals and patients involved

Type of tele visit	Case study	Visit description	Healthcare providers/depts.	Patients
Routine – structured	Cardiological consultation	These visits, for patients with implantable devices, are held at regular intervals (twice a year) intending to check the status of the device and discuss pre-determined issues.	1 Hospital dept	15 patients
Routine – Open-ended	Occupational health assessment	These visits take place every couple of years and they may lead to identifying unpredictable issues.	1 Occupational physician	15 employees
Acute – structured	Gestational diabetes monitoring	Visits are needed to evaluate, for a short period (1 trimester), predetermined parameters of pregnant women that develop diabetes and provide guidance regarding emerging needs.	1 Diabetes centre	15 patients
Acute – open-ended	Paediatrician visit	These consultations are triggered by an acute need and the clinical encounter cannot be pre-structured due to the multifariousness of the conditions that may affect children.	5 Paediatricians	50 patients (10 per paediatrician)

The theoretically informed choice of cases has a twofold objective. On the one hand, it allows identifying opportunities and challenges that may apply to similar patient–provider relationships. For instance, the case study of paediatricians may be used to gather a preliminary understanding of challenges and barriers to adoption in context where reasons for encounter are triggered by an acute need and vary from patient to patient, such as general practitioners and emergency medical services. On the other hand, through the generalisability of the results, it serves the purpose of conducting a quick evaluation of a technical solution which is required to be designed, implemented and scaled rapidly to provide a timely response to the pandemic.

The case studies employed a mixed-method approach utilising both qualitative and quantitative analytical techniques to investigate three main dimensions: organisational impact, modification of patient-provider relationship, acceptability and usability of the technical solution. The organisational impact was investigated involving all healthcare providers in two online focus groups, the first to establish a baseline before the onset of the project and a final one to discuss the results of the use of the platform. The modification of the patient–provider relationship was explored with specific questions in the focus group with providers and through dedicated telephone interviews with patients to have a narrative account of their experience and discuss in more detail the future of teleconsultation. The acceptability of the platform was assessed by asking all patients to complete a survey about their socio-demographic profile, the usability of TreC_Televisita (assessed through the System Usability Scale (Lewis 2018), the appreciation of relevant dimensions of the visit, and comments regarding the possibility to extend such form of communication beyond the coronavirus pandemic period.

The analysis of the initial focus groups with providers sheds some light on the perceived opportunities and barriers of TreC_Televisita. Healthcare providers have shown appreciation for the opportunity to test (and possibly adopt) an ‘official’ technology. While they appreciated the possibility to keep in contact with patients during the hardest months of the pandemic offered by SMS, instant messaging platforms (i.e. WhatsApp), email, Google Meet or the like, they hailed a system whose use was explicitly endorsed by the local healthcare trust. Moreover, such a system is perceived as instrumental to identify one preferred channel of communication with patients. Moreover, clinicians regarded with particular interest the control granted by the platform which, unlike other systems, offers the possibility to manage the flow of communication with patients allowing for a smoother integration of teleconsultation in their usual workflow.

In order to further scale up the solution to more patients and clinical domains, a strategy should be defined to overcome the potential barriers that may arise, as defined below.

### Ensuring the long-term sustainability of TreC_Televisita

Even before the number of people infected by COVID-19 started to dwindle and limitation restrictions were waived, TreC_Televisita was becoming a structural part of the services offered within TreC; therefore, the fourth step of the methodology relates to the identification of barriers that might undermine the future sustainability of the tool. This issue should be thoroughly considered in order to avoid what in literature is defined as ‘pilotitis’, i.e. ‘the growing concern that most of the telemedicine innovations never make the threshold from pilot project stage to full implementation and translation into quality care practice’ (Harst et al. 2019). Thus, ensuring the scalability and the long-term implementation of eHealth solutions requires an upstream definition of what the barriers are and the risks that such solutions might face as well as the strategies for their mitigation.

This section aims at identifying the main potential barriers for implementation of the TreC_Televisita solution in the long term. In the short term, TreC_Televisita aimed at its definition and implementation in the Trentino context to mitigate the effects of the COVID-19 pandemic; in the future, the solution will be applied to a broader context, be it more clinical domains or larger geographical focus in a sustainable way. In fact, a wider domain of application of tele visit services originated in Trentino Province foresees the potential engagement of other Italian public administrations through the practice of ‘re-use’ of services.^11^ In this regard, according to the implementation research guidelines (Peters et al. 2013), contextual differences are key factors leading to failure when transferring a service from one country to another. Hence, even though TreC_Televisita is not yet in the scalability phase, it was decided to conduct a revision of state-of-the-art frameworks on barriers to telemedicine implementation to analyse the applicability of potential solutions and lessons learnt to the Trentino context.

Therefore, while the authors acknowledge the numerous works related to the definition of the main barriers hampering the implementation of telemedicine solutions in different geographic contexts and with different clinical targets (e.g. Medhanyie et al. 2015 in Ethiopia for the implementation of telemedicine for maternal healthcare; Ag Ahmed et al. 2017 changed perspective by defining success factors for maternal health in Sub-Saharan Africa; Jang-Jaccard et al. 2014 in rural Australia; or Waterson et al. 2012 who described the barriers in the UK for solutions for frail elderly, just to mention a few), it was decided to consider only the literature review that proposes a barrier framework targeting telemedicine solutions without specific clinical or geographical reference. First, we analysed Harst et al. (2019) recent survey as a review of previous reviews; however, all the works considered present a form of verticalization, either clinical or geographical, often both. After analysing the list of Harst’s references, van Dyk’s review of telehealth services (2014) was thoroughly evaluated, especially where the author highlights the relationship between Tanriverdi and Iacono’s (1998) and Khoja’s (Khoja et al. 2007) works: his result, displayed in Table 2 below, is a framework where Tanriverdi and Iacono’s barriers are defined at micro as well as macro level and that matches the potential barriers identified for the implementation of telemedicine in Trentino.

**Table 2:** van Dyk’s framework (van Dyk 2014)

Barriers	Micro-level	Macro-level
Technical	Technology (hardware and software)	Technology (ICT infrastructure)
Behavioural	Learning (healthcare workers)	Society
Economical	Core (budget)	Policy (reimbursement models)
Organisational	Core (process integration and prioritization)	Policy (planning and promotion of telehealth)

van Dyk’s barrier framework is the one that better matches the Trentino healthcare context and thus was chosen for the analysis of the barriers related to the large-scale adoption of TreC_Televisita. The framework defines four macro categories that include the main aspects for its development (technical and economical) and first adoption (behavioural and organisational): the subdivision provides a definition in relation to the actors involved (end-users, policy, society as a whole) and to the practical elements necessary for its implementation. Moreover, for the subject of this paper, such a framework was extended with other criteria, namely the legal and ethical aspects involved in providing tele visit services, to better include all the dimensions of TreC_Televisita and to be more adaptable to the Trentino healthcare system context. Table 3 below describes the barriers that TreC_Televisita will/might need to overcome (or has already overcome) for its large-scale implementation. A set of different goals have been identified by the Healthcare Trust administration, considering the different stages of the epidemic. A short-term goal was to reach 30% of tele visits in the first part of the epidemic peak (on the total of clinical visits performed for that period), a target of 50% was set as a standard percentage considering follow up visits, and a long-term target of 60% was identified, including also tele visits performed by the community clinics at local districts level.

**Table 3:** TreC_Televisita barriers for large-scale implementation

Barriers type		TreC_Televisita potential barriers	TreC_Televisita possible strategies to overcome barriers
Micro level	Technical	Integration of third-party services (i.e. Zoom, wellbeing devices, etc.) Integration with third party medical devices (apps, glucometer, etc.) Integration with PHR Integration with appointment booking	TreC_Televisita has been arranged to be connected with third party services and devices, as well as with internal components, such as PHR and appointment booking service. This has been done as far as APIs and data access are concerned: in particular, session management will be coordinated between both data generated by external devices in addition to own data.
	Economical	Funding unavailable	An initial investment was needed to develop the service, which is based on the infrastructure already existing in the Healthcare Trust (TreC) and that needs basic equipment (PC). On the other hand, patients only need a smartphone/tablet/PC. At the beginning of the solution development, TreC_Televisita implementation was financed by the Fondazione Valorizzazione Ricerca Trentina (VRT) through a specific grant. In the long term, it has been recently declared that investments will be made by APSS to set up 20 clinics spread all over the territory; however, in case the budget available will not be enough to cover these expenses, other sources will be targeted for this purpose (e.g. European Commission funds).
	Behavioural (end-users)	Tele visit being perceived as a ‘lesser patient–provider interaction’ Healthcare staff is not trained for using the technology provided Healthcare staff is not prone to use the technology; need to integrate TreC_Televisita with the existing software and organisational workflow Privacy	Tele visit may not be the adequate solution for each and every consultation. In the upcoming months a strategy will be defined to identify the type of visits that patients deem necessary to be conducted in person (e.g. first visits). Within the Trentino Healthcare system there is a systematic approach to the training of healthcare staff: for example, at the local level there is the ‘Scuola di Formazione Specifica in Medicina Generale’; at the European level, within European projects, there is the Erasmus +^a^ ‘Training Blueprint for the Digital Transformation of Health and Care’ project aiming at increasing health professionals digital & soft skills. In addition to structuring training courses on digital knowledge for doctors and, in this way, increasing their awareness of these innovations, it is also important to make technology easier and helpful for clinical activity. That is to integrate data from devices and sensors (e.g. ECG, digital stethoscope, electronic scale, Fitbit) on the TreC platform in order to provide the doctors with all the information and data to carry out a complete evaluation of the patient remotely. Cardiologists and diabetologists stressed the importance of integrating the data acquired by monitoring sensors provided by vendors within the system. TreC_Televisita is compliant with very high standards, with particular attention to the European regulation on personal data (GDPR). In addition, it is important to make the doctor aware of the importance of the data and its conservation.
	Organisational	Cumbersome integration of tele visits into healthcare professionals’ work process. Resistance from healthcare professionals (out of their comfort zone) Some visits result difficult or impossible to deliver via tele visits (e.g. surgery, neurodegenerative diseases)	TreC_Televista became an integrated three-level service: before (1), during (2) and after (3) the consultation (thus patients can send information to their doctor in advance and can also be monitored after the consultation – through health reporting). Thus, tele visits are becoming much more complete and more effective compared to the past. Health care professionals can request an ad-hoc customisation of the remote consultation services to be adapted to their patients’ needs/diseases and ask for specific requirements. The 20 clinics that will be set up will be equipped with cutting-edge technology to support the implementation of tele visit, trying to bridge the distance divide.
Macro level	Technical	Missing integration with Trentino healthcare infrastructure	TreC_Televisita has been implemented from the start as part of the TreC infrastructure, which is the platform serving the whole Trentino citizenship.
	Economical	Reimbursement	The PAT decided that, during the sanitary emergency, tele visits would benefit from the exemption of sharing health care expenses (ticket) for all patients registered with the national health service (limited to the duration of the state of emergency) owing to the Council resolution n°465 of 2020 on ‘Provisions on Telemedicine and other provisions to deal with the emergency from COVID-19’. In the long-term, the envisioned solution should consider how to include tele visit as a prescribed service of the local health system and the associated degree of reimbursement.
	Behavioural	People needing assistance that cannot use the service autonomously (e.g. low digital literacy, disabled, language barrier)	In the next months, the tele visit service will be supported by social services/nurses that will assist people directly at their home (assisted tele visit). In the long-term, in the 20 clinics nurses will support patients during tele visits.
	Organisational	Policy will not support the adoption of the service. Tele visit are not integrated in the overall processes defined for healthcare provisioning in the Province	TS4.0 strategy and decisions are planned in common agreement between its three main stakeholders, i.e. the Autonomous Province of Trento – PAT (the policy-maker) the Healthcare Trust – APSS and the Bruno Kessler Foundation – FBK (the technological innovator): this ensures that the decision implemented by APSS and FBK are fully supported and promoted by PAT. APSS will conduct a change management strategy based on implementation research to incorporate the tele visit service as shown in Fig. 2.

^a^http://trainblue.eu/

Even though the implementation of a tele visit service poses major barriers and challenges that require a great effort from the decision makers, significant opportunities arise as well. First of all, during the COVID-19 emergency TreC_Televisita has enabled clinicians and patients to respect the social distancing measures, decreasing the risk of contagion while performing healthcare services; at the same time, tele visit allowed eliminating geographical barriers among patients and healthcare professionals. In addition, the scale-up of TreC_Televisita might allow a reduction of costs in the long term. As a matter of fact, the European Union has encouraged the development of telemedicine throughout the years, with guidelines, communication, action plans, as well as financial investments. In 2008, the European Commission adopted the ‘Communication on telemedicine for the benefit of patients, healthcare systems and society^12^’, defining three goals: (i) to build confidence in and acceptance of telemedicine, (ii) clarify legal aspects and (iii) solve technical issues and ease market development. The COVID-19 emergency was a strong trigger that led healthcare providers and policy-makers towards this direction: the reasons why decision makers did not commit to the implementation of tele visits before might be connected to the difficulties in assessing the efficacy of telehealth. In fact, this depends on multiple factors (e.g. demographics, quality and modality of the service provided) and, therefore, the scientific community is still evaluating its pros and cons. In this perspective, after a few years of implementation at operating speed, TreC_Televisita might be a forerunning experience that will allow a proper assessment of clinical, social, organisational and economic factors, eventually leading to an increasing number of tele visit services to become structural in other healthcare systems.

## Results

This section describes the results achieved after the TreC_Televisita project development and implementation. The results are presented taking into consideration the outcomes of each of the four methodological steps described in section 3.

i. The organisational and technical requirements elicited in the first step were analysed with an ad-hoc evaluation that was carried out to investigate the core organisational aspects as well as the perceived utility by using a mixed-method approach (focus groups, interviews and questionnaires for both doctors and patients).

From an organisational viewpoint, within the COVID-19 context the possibility to provide telemedicine services helped in the daily organisation of the workload. Some clinicians could do smart working, the offices were free and available for urgent, non-postponable visits, and the time for hygienisation between visits was reduced. Beyond the pandemic, the pilots remarked the importance of organisational changes for a new technological approach to be successful. The introduction of TreC_Televisita, in particular in the medical areas involving chronic diseases (i.e. cardiological consultation and diabetes monitoring), encouraged a shift from the traditional periodic visits to on-demand visits, where the patient is constantly remotely monitored and it is called for a face-to-face visit with the clinician only when a visit is needed, in a proactive way. In addition, particularly for occupational health visits, the adoption of tele visits was perceived as a viable tool enabling a significant decrease in terms of workload of health professionals on one side, and in terms of interfering with workers’ agenda on the other.

In terms of technical acceptability, the evaluation highlighted a substantial acceptability of the tele visit platform in all the evaluated settings. The patients and clinicians that responded to the survey (respectively, 34 and 9) defined the application easy to use, essential and user friendly. None of the interviewed participants complained about the usability of the application, whilst approximately 80% of the respondents described the procedure as ‘easy’. The level of satisfaction was high for nine respondents out of ten, yet the internet connection quality represented a critical aspect for those patients living in the rural area of the Province (almost one third of the participants). At the end of the pilots, several suggestions for improving the system in terms of functions were collected. Even though the sample size does not allow to make strong claims about the acceptability of the system, most of the collected feedback suggests a positive trend in the technical acceptability of the platform. In all cases, the tele visits were used to carry out check-ups, follow-up visits and not as a first evaluation of the patient.

ii. The validity assessment of the tele visit part of the second step of the methodology, the overall quality and the comprehensiveness of a face-to-face visit at the clinic is still not comparable with a tele visit for all the four areas investigated. Nonetheless, this latter has been perceived as an extremely useful tool and it has proved to be acceptable and feasible for both the medical staff and the patients that agreed to do a tele visit. The privacy by design approach implemented successfully allowed the development of a tool compliant with GDPR, and no data breach cases were reported, nor inquiries from the data subjects were received.
iii. The piloting of TreC_Televisita was implemented from April to October 2020, targeting cardiological consultation, occupational health assessment, gestational diabetes monitoring and paediatrician visits. These areas were identified considering the various organisational structures of the services, their availability and the strategic asset in terms of telemedicine. Before implementing the actual piloting, a preliminary phase was dedicated to the analysis of the needs and an ad-hoc design and integration of the required functionalities. During the piloting phase, five paediatricians, two diabetologists, one cardiologist and one occupational MD were involved.
  When considering the behavioural component, it should be underlined that clinicians were free to decide which patients were to be involved in the piloting process. The patients (or their parents, in case of paediatric patients) enrolled for the tele visits, the average group was composed of patients 41–48 years old. The majority of the sample used a smartphone as the tele visit device rather than a tablet (80% versus 20% approximately), whilst the duration of the remote tele visit ranged from 10 to 20 min in the majority of the cases (70% roughly). Generally, patients reported that they could understand the doctor well and that they were able to speak with the healthcare staff for a reasonable and satisfactory period of time.
iv. The results mentioned in (i), (ii) and (iii) above are directly linked with a potential scale-up of the tele visit as they match the van Dyk’s (2014) framework used for the definition of the barriers to the large-scale adoption of TreC_Televisita (Table 3). In particular, the technical issues are addressed mainly in (i), the organisational dimension is covered in all the three steps and the behavioural aspects are addressed in (iii). With regard to the economic issues considered in the evaluation, this was not investigated due to the lack of economic indices. Nevertheless, it can be argued that patients achieved savings, particularly those living in rural areas of the region, in terms of journey and parking costs, general resources and time spent for the visit.

Overall, during the implementation of the initiative and the related evaluation phase, a number of positive implications of adopting a tele visit approach have been highlighted, namely: the possibility of avoiding unnecessary travel and contacts particularly during the COVID-19 epidemic; the possibility of limiting an organisational burden linked to visiting patients for simple routine follow-up visits at the clinic; the potential scalability of the tele visits approach within a broader stepped-care management, ranging from remote visits to face-to-face visit at the clinic when appropriated.

## Discussion and lessons learnt

The unexpectedness of the emergency has vividly exposed the whole Trentino system, with its strengths and weaknesses. While still facing the COVID-19 pandemic, it is now possible to identify several lessons learnt on the quick implementation of a tele visit service to mitigate the effects of the pandemic on the overall healthcare service provisioning.

First and foremost, it should be underlined that TreC_Televisita has been implemented in a critical context, when policy, research and clinicians were dealing with an unprecedented situation whose consequences have an impact both in the short and the long term. In the short term, the pandemic has boosted the implementation of remote services for patients. TS4.0 had already defined services such as TreC_Diabetes, TreC_Cardio and TreC_Oncologia for the management of chronic diseases with the support of ICTs, where a telemonitoring/tele visit model was preliminarily piloted and validated within specific medical specialities. The need for social distancing and the mobility restrictions have accelerated the definition and realisation of a further step in this direction, which is the realisation of tele visit services. In the long term, TreC_Televisita has highlighted that the introduction of a new healthcare service is more efficient when the whole system is underpinned by a precise design, especially from the organisational viewpoint. Figure 2 shows a sequence of subprocesses that are part of a wider system: when each step is clearly framed and the related inputs and outputs are defined, then the healthcare management can replace the subprocesses with more efficient ones or can decide whether to remove or add new ones, without jeopardising the final outcome, i.e. improving citizens’ health. Furthermore, in terms of sustainability, the possibility to frame each step on its own makes the cost-benefit analysis as well as the comparison with other solutions easier. In other words, how the process of service provisioning is structured, i.e. the design of the process, has been found to play a key role in the positive management of the healthcare service and defines its ability to quickly react to sudden events as well as the assessment of its sustainability. The pandemic has exposed the fragility of healthcare systems and has underscored the need for flexible intervention models able to cope with emergencies of different kinds, be it a pandemic, an earthquake, or others, adaptable enough to include new services that allow the whole system to keep running.

TS4.0 operates in the field of public health and, more specifically, it is coherent with the implementation research as it combines diverse perspectives from different fields, namely policy, research and clinical perspectives. By including a governance model encompassing these three complementary perspectives, TS4.0 constitutes a regional strength by aligning the different priorities and agendas towards common goals. This kind of institutional territorial alignment is seen as a good practice transferable to other European regions to accelerate the provisioning of quality services to the population. One important element that has strengthened TS4.0 impact, is the existence of a common healthcare provisioning technological platform, TreC, which has allowed the development of new, jointly designed services. Another factor that reinforced the relationship is the organisational structure that has been developed, with a core management body, the Steering Committee, associated with an Executive Committee and a Project Management Board; this has allowed the systematic cooperation of the three institutions at different levels creating first an exchange and then a new body that has proven to be solid enough to cope with emergency situations and positively face them.

Particularly in the context of the COVID-19 emergency, the above-mentioned organisational and technical assets played a fundamental role in the quick identification of possible solutions and actions to mitigate the effects of the pandemic. In addition, other unforeseen lessons learnt were achieved such as:

- to set up a flexible healthcare provisioning process at the basis of a sustainable approach towards emergency management;
- to address the emergency while in parallel considering the barriers for implementation of a long-lasting sustainable solution;
- to focus on multi-factor issues for the implementation of the solution (technical, organisational, regulatory, etc.);
- to conduct quick, incremental and iterative processes for implementing and validating the introduction of innovations for managing emergency situations;
- to involve the target stakeholders and users for early design and validation phases (policy, research, clinicians, patients);
- to define social and healthcare integrated solutions to increase the accessibility to healthcare services to people with difficulties in autonomously using it (e.g. because of a low level of digital literacy).

## Conclusions

The COVID-19 pandemic urgently called upon the need of a service based on the remote relationship between clinicians and patients; however, such shift requires structural changes in the healthcare provisioning as well as solutions that can positively overcome major barriers ensuring scalability and long-term adoption. In this perspective, this paper highlights the genesis and main features of the TreC_Televisita solution as an integrated service into the Trentino healthcare continuum and as a technological solution, part of the pre-existing TreC infrastructure, and in the regulatory context where it operates (i.e. the Trentino healthcare system as a whole – legal, ethical, clinical, etc., points of view).

To the best of our knowledge, TreC_Televisita represents an exemplar innovation in the panorama of the quick introduction of telemedicine services in Italy, considering how it is fully integrated into the provincial healthcare ecosystem from the organisational, regulatory and technological viewpoints. The results of the piloting phase have shown that TreC Televisita is a viable and acceptable approach to promote telemedicine in the context of the COVID-19 pandemic and beyond. Even if tested on a limited number of medical areas, the representativeness of the selected settings could be considered as adequate, given their diverse organisational assets. TreC_Televisita was largely based on a series of former research projects and pilots implemented in the Province of Trento as part of a larger telemedicine strategy; then, it evolved rapidly, and will continue evolving in the next months and years to become a structural pillar of the healthcare system in Trentino. So far, the service has been used for 30% of the total visits performed in the Province. The medium-term objective for 2021 is to maintain this percentage by expanding tele visits to other specialities within the provincial healthcare system and so this percentage will be the average among different domains. In fact, while some specialities can be more suitable for performing tele visits (e.g. chronic diseases), others are less apt because of current technological limitations or specific tests to be performed, such as general surgery where the surgeon needs to conduct face-to-face operations (e.g. palpate the patient’s abdomen). In parallel with the aim to expand tele visits across different domains, it is planned to engage a wider portion of the population by employing social services to assist people unable to perform tele visits autonomously at their home, i.e. assisted tele visits where a nurse or social assistant can support the patient to conduct the tele visit (e.g. patients with physical or cognitive disabilities). Furthermore, the long-term goals are even more ambitious as the will is to reach 60% of tele visits on the total provided; some substantial investments are planned with this purpose for the setup of 20 clinics spread all over the territory. This may expand tele visit to people with specific conditions (e.g. older adults, people with cognitive impairment, low digital-literacy patients) as nurses will support them; successively, it will increase the quality of the visits as these clinics will be equipped with cutting-edge ICT medical devices (e.g. electronic stethoscope) that will further increase the quality and efficiency of tele visits, providing more accurate parameters to the specialist.

## Data Availability

Not applicable.

## Footnotes

1 Free translation of the author STE from Ministero della Salute, Telemedicina, Linee di indirizzo nazionali, http://www.salute.gov.it/imgs/C_17_pubblicazioni_2129_allegato.pdf

2 https://trecovid.apss.tn.it/app/tutorial

3 Further information can be found in this video presented during the Trento Film Festival: https://www.youtube.com/watch?v=-mpmLjj7jAE&t=223s

4 Resolution of the provincial council n. 15,047/2006 most recently amended by resolution no. 1969/2019

5 https://edpb.europa.eu/sites/edpb/files/files/file1/edpb_guidelines_202003_healthdatascientificresearchcovid19_en.pdf

6 https://ec.europa.eu/info/sites/info/files/5_en_act_part1_v3.pdf

7 https://www.garanteprivacy.it/documents/10160/0/Raccolta+delle+principali+disposizioni+adottate++in+relazione+allo+stato+di+emergenza+epidemiologica+da+Covid-19+aventi+implicazioni+in+materia+di+protezione+dei+dati+personali+%28AGGIORNATO+AL+30+LUGLIO+2020%29.pdf/a3c13c1b-f14a-2cb3-c63b-d65dce1df8b7?version=11.0

8 https://eur-lex.europa.eu/eli/reg/2016/679/oj

9 https://portale.fnomceo.it/fnomceo-alle-regioni-su-telemedicina-rinviare-esame-del-provvedimento-e-convocare-tavolo-permanente-congiunto/

10 >http://www.quotidianosanita.it/allegati/allegato4648608.pdf

11 https://www.agid.gov.it/it/design-servizi/riuso-open-source/linee-guida-acquisizione-riuso-software-pa

12 https://ec.europa.eu/transparency/regdoc/rep/1/2008/EN/1-2008-689-EN-F1-1.Pdf

## Code availability

TreC_Televisita is a service part of the TreC platform, which is the Personal Health Record of the Trentino Healthcare Trust, Azienda Provinciale per i Servizi Sanitari. The whole TreC platform, and thus the whole TreC_Televisita software, is available under the open API specification available at https://www.openapis.org.
